# A new species of *Calamaria* (Squamata, Calamariidae) from southern China, previously confused with *Calamaria pavimentata* Duméril, Bibron & Duméril, 1854

**DOI:** 10.3897/zookeys.1277.187107

**Published:** 2026-04-17

**Authors:** Shuo Qi, Tan Van Nguyen, Jian-Huan Yang, Yu-Hao Xu, Patrick David, Jing-Song Shi, Jing-Jian Liu, Can-Zhong Rong, Alexey M. Korolev, Nikolay A. Poyarkov, Ying-Yong Wang

**Affiliations:** 1 School of Ecology / School of Life Sciences, Sun Yat-sen University, Guangzhou 510275, China Institute of Vertebrate Paleontology and Paleoanthropology, Chinese Academy of Sciences Beijing China; 2 The School of Medicine & Pharmacy, Duy Tan University, Da Nang, 550000, Vietnam School of Ecology / School of Life Sciences, Sun Yat-sen University Guangzhou China; 3 Center for Entomology & Parasitology Research, Duy Tan University, Da Nang, 550000, Vietnam Department of Vertebrate Zoology, Lomonosov Moscow State University Moscow Russia; 4 Kadoorie Conservation China, Kadoorie Farm and Botanic Garden, Hong Kong 999077, China Institut de Systématique, Évolution et Biodiversité (ISYEB), Muséum National d’Histoire Naturelle, Sorbonne Université, École Pratique des Hautes Études, Université des Antilles Paris France; 5 State Key Laboratory of Plateau Ecology and Agriculture, Qinghai University, Xining 810016, China State Key Laboratory of Plateau Ecology and Agriculture, Qinghai University Xining China; 6 Institut de Systématique, Évolution et Biodiversité (ISYEB), Muséum National d’Histoire Naturelle, Sorbonne Université, École Pratique des Hautes Études, Université des Antilles, CNRS, CP 30, 57 rue Cuvier, F-75005 Paris, France The School of Medicine & Pharmacy, Duy Tan University Da Nang Vietnam; 7 Institute of Vertebrate Paleontology and Paleoanthropology, Chinese Academy of Sciences, Beijing 100044, China Center for Entomology & Parasitology Research, Duy Tan University Da Nang Vietnam; 8 Guangzhou Xiaogangwan Primary School, Guangzhou 510220, China Kadoorie Conservation China, Kadoorie Farm and Botanic Garden Hong Kong China; 9 Shenzhen Mangrove Wetlands Conservation Foundation, Shenzhen 518034, China Guangzhou Xiaogangwan Primary School Guangzhou China; 10 Department of Vertebrate Zoology, Lomonosov Moscow State University, Leninskiye Gory, GSP–1, Moscow 119234, Russia Shenzhen Mangrove Wetlands Conservation Foundation Shenzhen China

**Keywords:** *Calamaria similis* sp. nov., Guangdong, Guangxi, integrative taxonomy, morphology, mtDNA, species complex

## Abstract

*Calamaria pavimentata* Duméril, Bibron & Duméril, 1854 was originally described from Java Island, Indonesia, yet specimens from southern China, particularly Guangxi, have long been referred to this species based on general morphological resemblance. Herein, the taxonomic status of Chinese populations previously referred to as *Calamaria pavimentata* is re-evaluated using an integrative approach combining morphological data and mitochondrial DNA analyses, based on four specimens from Yangjiang City, Guangdong Province, and Chongzuo City, Guangxi Zhuang Autonomous Region, China. Phylogenetic analyses recover the Chinese specimens as a distinct and well-supported lineage within *Calamaria*. Notably, the Guangdong and Guangxi populations exhibit a relatively high intraspecific mitochondrial divergence (uncorrected *p*-distance = 10.78% based on the cytochrome *b* gene), despite the absence of consistent diagnostic morphological differences. Although mitochondrial DNA data from topotypic *C. pavimentata*, are currently unavailable, the observed morphological differences, together with the pronounced geographic disjunction between Java and southern China, support the recognition of the Chinese population as a distinct species, herein described as *Calamaria similis***sp. nov**. from Guangdong and Guangxi, China. Detailed morphological examinations reveal that *Calamaria similis***sp. nov**. differs from *C. pavimentata* and all of its currently recognized synonyms by having higher ventral scale counts in females, fewer subcaudal scales in males (but slightly more in females), a smaller maximum total length in males, and a shorter relative tail length in both sexes. Our results highlight the need for renewed field surveys in Java Island, Indonesia to rediscover *C. pavimentata* sensu stricto, and emphasize that other populations previously identified as *C. pavimentata* should be re-evaluated using integrative taxonomic approaches.

## Introduction

The genus *Calamaria* H. Boie in F. Boie, 1827, comprises a group of small, semi-fossorial snakes widely distributed from northeastern India through mainland Southeast Asia to the Greater Sunda Islands and the Philippines, several species pass Wallace’s line and inhabit Sulawesi and the Maluku Islands (Boie 1827; Inger and Marx 1965; de Lang and Vogel 2005; de Lang 2013; David et al. 2023; Uetz et al. 2026). Members of the genus are characterized by a cylindrical, vermiform body; a short, thick tail; indistinct demarcation between head and neck; small eyes with round pupils; reduced head scalation lacking internasals, loreals, and temporals; four or five supralabials, with the posterior one broadly contacting the parietal; a large paraparietal scale; smooth dorsal scales arranged in 13 rows throughout the body; a single cloacal plate; and paired subcaudal scales (Inger and Marx 1965; David et al. 2023). Owing to their secretive habits and conservative external morphology, species boundaries within *Calamaria* have long been considered difficult to assess.

Among the most taxonomically problematic taxa within the genus is *Calamaria pavimentata* Duméril, Bibron & Duméril, 1854. This species was originally described from Java Island, Indonesia, a type locality that has long been regarded as doubtful because no verified records of *C. pavimentata* are known from the island (Inger and Marx 1965). Despite this uncertainty, *C. pavimentata* has traditionally been treated as a widely distributed species, reported from southern China, as well as from mainland Southeast Asia, northeastern India, and eastern Bangladesh (e.g., Zhao et al. 1998; Poyarkov et al. 2023; Rahman et al. 2026). The application of this name to geographically distant populations has been based largely on general similarities in body form and scalation, rather than on rigorous integrative analyses.

Recent taxonomic studies have demonstrated that several populations previously referred to *Calamaria pavimentata* actually represent distinct evolutionary lineages, including *Calamaria annamensis* Bourret, 1937, *C. arcana* Yeung, Lau & Yang, 2022, *C. berezowskii* Günther, 1896, *C. mizoramensis* Lalremsanga, Malsawmdawngliana, Bal, Vabeiryureilai, Hruaia, Korolev, Vogel, Poyarkov & Nguyen, 2026 and *C. synergis* Zhang, Xu, Nguyen, Poyarkov, Vogel, Wang & Huang, 2025 (see Yeung et al. 2022; Liang et al. 2024; Zhang et al. 2025a; Lalremsanga et al. 2026; Korolev et al. 2026). These taxonomic advances have largely been achieved through integrative approaches combining detailed morphological comparisons with mitochondrial DNA data, which have led to the recognition of previously unrecognized diversity and the redefinition of species boundaries, particularly in mainland China and adjacent regions (e.g., Yang and Zheng 2018; Yeung et al. 2022; Liang et al. 2024; Zhang et al. 2025a). Collectively, these findings indicate that the traditional concept of *C. pavimentata* likely encompasses multiple cryptic or morphologically similar species. In light of these findings, we consider *C. pavimentata* to be a species complex, and the status of several nominal taxa and junior synonyms historically associated with *Calamaria pavimentata* warrant careful re-evaluation based on comprehensive morphological and molecular data. A summary of the taxonomic history and type localities involved is presented in Table 1.

**Table 1.** Species-level scientific names erected for members of the *Calamaria pavimentata* species complex. Notes: ? = requires verification.

Authority	Original taxon name	Type locality	Present taxonomy	Proposed taxonomy
Duméril et al., 1854	*Calamaria pavimentata*	Java, Indonesia	*Calamaria pavimentata*	*Calamaria pavimentata*
Duméril et al., 1854	*Calamaria quadri-maculata*	Java, Indonesia	synonym of *C. pavimentata*	synonym of *C. pavimentata*
Günther, 1864	*Calamaria siamensis*	Luangphabang Range in north-western Laos	synonym of *C. pavimentata*	synonym of *C. pavimentata*^?^
Günther, 1896	*Calamaria berezowskii*	Pingwu, Sichuan, China	*Calamaria berezowskii*	*Calamaria berezowskii*
Stejneger, 1901	*Calamaria pfefferi*	Miyako-jima Is, Yaeyama, Ryukyu, Japan	*Calamaria pfefferi*	*Calamaria pfefferi*
Smith, 1921	Calamaria pavimentata var. uniformis	Langbian Plateau, Lam Dong, Vietnam	synonym of *C. pavimentata*	synonym of *C. pavimentata*^?^
Maki, 1931	*Calamaria pavimentata formosana*	Mt. Ali, Chiayi, Taiwan, China	synonym of *C. pavimentata*	synonym of *C. pavimentata*^?^
Bourret, 1934	*Calamaria pavimentata banaensis*	Ba Na-Nui Chua NR, Da Nang, Vietnam	synonym of *C. pavimentata*	synonym of *C. pavimentata*^?^
Bourret, 1937	*Calamaria pavimentata annamensis*	Bac Huong Hoa NR, Quang Tri, Vietnam	*Calamaria annamensis*	*Calamaria annamensis*
Takara, 1962	*Calamaria pavimentata miyarai*	Yonaguni-jima Is, Yaeyama, Ryukyu, Japan	synonym of *C. pavimentata*	synonym of *C. pavimentata*^?^
Yeung et al., 2022	*Calamaria arcana*	Mt. Dadongshan, Qingyuan, Guangdong, China	*Calamaria arcana*	*Calamaria arcana*
Zhang et al., 2025	*Calamaria synergis*	Mt. Jinuo, Jinghong, Xishuangbanna, Yunnan, China	*Calamaria synergis*	*Calamaria synergis*
Lalremsanga et al., 2026	*Calamaria mizoramensis*	Reiek, Mamit, Mizoram, India	*Calamaria mizoramensis*	*Calamaria mizoramensis*
This study	*Calamaria* “*pavimentata*”	Yongshui, Shuangjia, Yangchun, Yangjiang, Guangdong, China	*Calamaria* “*pavimentata*”	*Calamaria similis* sp. nov.

**Figure 1.**
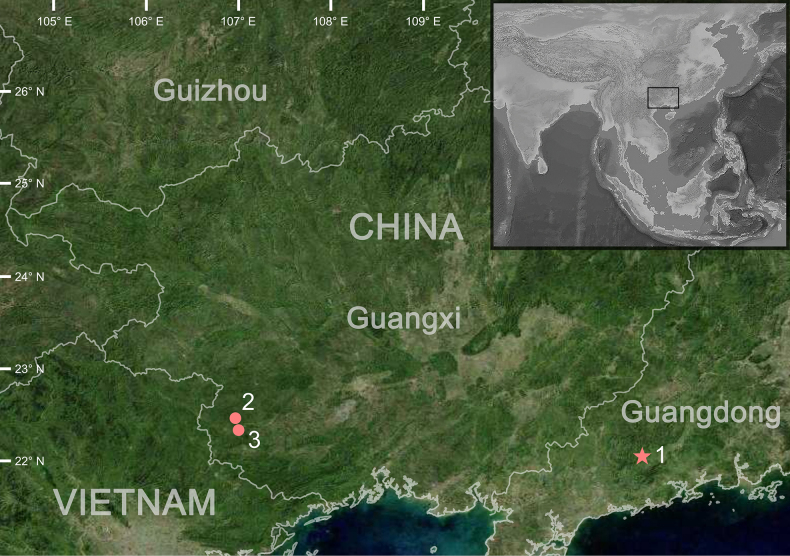
Map showing the distribution of the *Calamaria similis* sp. nov. The star indicates the type locality. White numbers indicate collecting localities: 1. Dahe Village, Shuangjiao Town, Yangchun City, Yangjiang City, Guangdong Province, China; 2. Nonggang National NR, Longzhou County, Chongzuo City, Guangxi ZAR, China; 3. Shangjin Village, Shuikou Town, Longzhou County, Chongzuo City, Guangxi ZAR, China.

In southern China (see Fig. 1), some *Calamaria* specimens from Guangdong (including Yangjiang City) and Guangxi (including Chongzuo City) have consistently been identified as *C. pavimentata* in the literature, yet their taxonomic status has never been critically evaluated (e.g., Fan 1931; Zhao et al. 1998; Yang and Zheng 2018; Zhang et al. 2025a). Preliminary observations suggest these populations exhibit consistent differences in ventral and subcaudal scale counts and body proportions when compared with “true” *C. pavimentata* described in the original description and subsequent taxonomic accounts. In addition, the substantial geographic disjunction between Java—the putative type locality of *C. pavimentata* and southern China raises further doubts regarding the conspecificity of these populations. Therefore, through the integration of morphological comparisons, molecular phylogenetic analyses, and geographic distribution information, this study describes the southern Chinese populations long identified as *Calamaria pavimentata* as a new species. The aim is to provide a more systematic assessment of the taxonomic status of *Calamaria* in southern China and to contribute to species delimitation within this genus.

## Materials and methods

### Specimen collection and examination

**Figure 2.**
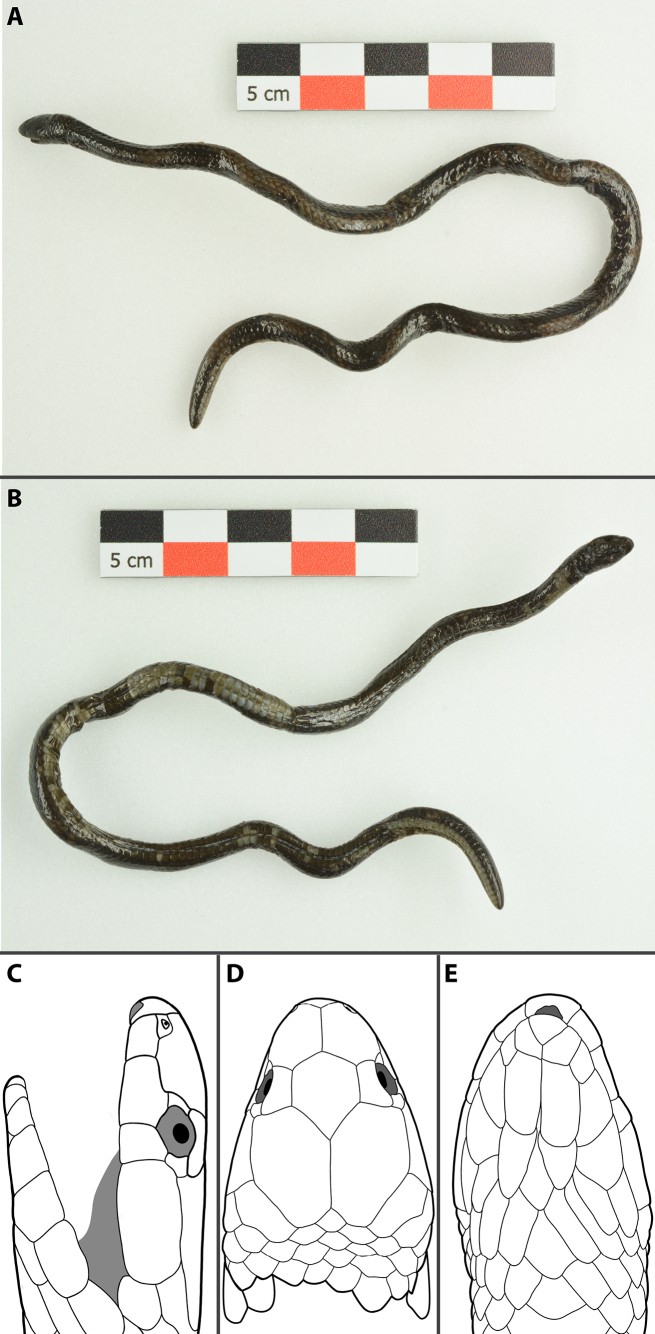
*Calamaria pavimentata*, adult male (MNHN-RA-3298, holotype), in preservative. **A**. General dorsal view; **B**. General ventral view; **C**. Lateral view of the head; **D**. Dorsal view of the head; **E**. Ventral view of the head. Photographs by J. Courtois (**A, B**). Line drawings by AMK (**C–E**), not to scale.

We collected four specimens of *Calamaria* from Yangjiang City, Guangdong Province and Chongzuo City, Guangxi Zhuang Autonomous Region, China. Following capture, the specimens were euthanized with tricaine methanesulfonate (MS-222), fixed in 4% methanal, and finally preserved in 75% ethanol at approximately 25 °C for morphological examination. The liver or muscle tissues were excised prior to fixation and stored in 95% ethanol at -40 °C as tissue samples for molecular experiments. Specimens were deposited in the herpetological collections of the Museum of Biology, Sun Yat-sen University (**SYS**), Guangdong, China and the Kadoorie Farm and Botanic Garden (**KFBG**), Hong Kong, China. In addition, we examined the holotype (MNHN-RA-0.3298) of *Calamaria pavimentata*, currently housed in the herpetological collection of the Muséum national d’Histoire naturelle (**MNHN**), Paris, France (Fig. 2).

### Molecular analyses

Total genomic DNA was extracted from liver tissue samples using the TIANamp Genomic DNA Kit (Tiangen Biotech Co., Ltd, Beijing). A fragment of the mitochondrial cytochrome *b* (cyt *b*) gene was amplified using the primer pair L14910 (5’–GACCT-GTGATMTGAAAACCAYCGTTGT-3’) and H16064 (5’–CTTTGGTTTACAA-GAACAATGCTTTA-3’) following Burbrink et al. (2000). PCR amplification was run using the following cycling conditions: initial denaturing step at 94 °C for 5 min; followed by 35 cycles of 94 °C for 30 s, 48 °C for 1 min and 72 °C for 70 s; and final extension step at 72 °C for 10 min. PCR products were purified with spin columns and then sequenced with forward primers using BigDye Terminator Cycle Sequencing Kit as per the guidelines on an ABI Prism 3730 automated DNA sequencer by Guangzhou Tianyi Huiyuan Bio-tech Co., Ltd.

The dataset of calamariid snakes comprised the sequences of the cyt *b* gene of three specimens of *Calamaria* sp. from Guangdong and Guangxi, China, a cyt *b* sequence of KFBG 14507 (a specimen from Guangdong previously referred to as *C. pavimentata* by Yang and Zheng 2018; GenBank accession number MH445957), 46 congeneric sequences (including representatives of 16 nominal *Calamaria* species) and three outgroups (following Korolev et al. 2026), including *Orientocoluber spinalis* (Peters), *Elaphe quatuorlineata* (Lacépède), and *Lycodon rufozonatus* (Cantor), which were obtained from the NCBI database GenBank (see Table 1). We initially aligned nucleotide sequences in MAFFT online (Katoh et al. 2019) with default parameters and subsequently checked them by eye in BioEdit 7.0.5.2 (Hall 1999) and adjusted when required. Protein-coding sequences were translated into amino acids to confirm that no pseudogenes had been amplified. The mean uncorrected genetic *p*-distances between cyt *b* sequences of *Calamaria* were calculated with MEGA 6.0 with the pairwise deletion option (Tamura et al. 2013). The best-fit substitution models for the data set were selected for genes and codon positions using Partitionfinder 2.1.1 (Lanfear et al. 2016) based on the Akaike information criterion (AIC), which selected GTR+I+G for the first codon position, HKY+I for the second codon position, and GTR+G for the third codon position of the cyt *b* gene.

We inferred the matrilineal genealogy of *Calamaria* using Bayesian inference (BI) and maximum likelihood (ML) approaches. We used the IQ-TREE (Nguyen et al. 2015) online server (Trifinopoulos et al. 2016) to generate the ML tree and assessed the confidence in tree topology using 1,000 ultrafast-bootstrap replications (UFBS; Minh et al. 2013). We conducted BI using the command-line version of MrBayes 3.1.2 (Huelsenbeck and Ronquist 2001). Both ML and BI trees used the same substitution models inferred from PartitionFinder. Metropolis-coupled Markov chain Monte Carlo (MCMCMC) analyses were run for the BI analysis with one cold chain and three heated chains for 10 million generations and sampled every 1,000 generations. The run was checked to ensure the effective sample sizes (ESS) were all above 200 by exploring the likelihood plots using TRACER v. 1.7 (Rambaut et al. 2018). The first 1,000 trees (corresponding to 10% of the sampled trees) were discarded as burn-in. We assessed the confidence in tree topology by the posterior probability (PP) of the nodes (Huelsenbeck and Ronquist 2001). We a priori regard nodes with UFBS values of 95% or higher and PP values over 0.95 as strongly supported; UFBS values between 95% and 90% and PP values between 0.95 and 0.90 were regarded as well-supported, and lower values were regarded as a lack of node support (Minh et al. 2013).

### Morphological analysis

Terminology and measurements follow Inger and Marx (1965) and Poyarkov et al. (2019). Measurements were taken with a digital slide caliper to the nearest 0.1 mm, except for body and tail lengths, which were measured to the nearest millimeter with a measuring tape. The number of ventral scales was counted according to Dowling (1951). The numbers of dorsal scale rows are given at one head length behind the head, at midbody, and at one head length before the vent. The sex was determined by dissection (inspection of gonads) and/or by tail shape when dissection was not possible. Maxillary teeth of the specimens were counted based on the three-dimensional reconstructed model.

The following measurements (all in mm) and counts were taken: snout-vent length (**SVL**); tail length (**TaL**); total length (**TL**); ratio of tail length/total length (**TaL/TL**); anterior dorsal scale rows (**ASR**); dorsal scale rows at midbody (**MSR**); posterior dorsal scale rows (**PSR**); supralabial scales (**SL**); number of supralabials touching the eye (**SL-E**); infralabial scales (**IL**); preocular scales (**PrO**); postocular scales (**PoO**); subcaudal scales (**SC**); ventral scales (**VEN**). Asymmetric characters are given in left/right order.

Morphological characters of the examined specimens were compared in detail with those of other *Calamaria* species known from mainland China and the Indo-Burma Region. For comparative purposes, we relied on previously published data (e.g., Duméril et al. 1854; Inger and Marx 1965; Darevsky and Orlov 1992; Ziegler and Le 2005; Ziegler et al. 2007; Ziegler et al. 2008; Orlov 2009; Orlov et al. 2010; Yang and Zheng 2018; Poyarkov et al. 2019; Ziegler et al. 2019; Lee 2021; Yeung et al. 2022; Cai et al. 2023; Liang et al. 2024; Nguyen et al. 2025; Zhang et al. 2025a, 2025b; Korolev et al. 2026; Lalremsanga et al. 2026). Other abbreviations: **Mt**. = Mountain; **NR** = Nature Reserve; **NP** = National Park; **asl** = above sea level; **ZAR** = Zhuang Autonomous Region.

### Osteological examination

To study the skeletal morphology of *Calamaria* sp., we performed detailed micro-CT scanning for a single specimen, SYS r001816. The X-ray scanning was carried out with Nano-computerized tomography. Specimens were scanned using a GE v|tome|x m dual tube 300/180kV system in the Key Laboratory of Vertebrate Evolution and Human Origins, Institute of Vertebrate Paleontology and Paleoanthropology (IVPP), Chinese Academy of Sciences, China. The specimen was scanned with an energy beam of 80 kV and a flux of 80*μA using a 360° rotation and then reconstructed into the 4096*4096 matrix of 1536 slices. The final CT reconstructed skull images were exported with a minimum resolution of 4.137 μm. The skull images were exported from the virtual 3D model which was reconstructed by Volume Graphics Studio 3.0.

## Results

### Sequence variation

The final alignment of the cyt *b* mtDNA gene fragment contained 1,109 aligned nucleotides, of which 564 sites were conserved, 545 sites were variable, and 470 were parsimony informative. The transition-transversion bias (R) was estimated at 5.12. Nucleotide frequencies were 31.71% (A), 28.56% (T), 28.99% (C), and 10.74% (G) (all data are given for the ingroup only). We deposited the newly obtained sequences in GenBank under the accession numbers PZ158841–PZ158843 (see Table 2).

**Table 2.** DNA sequences, voucher specimens, and GenBank accession numbers of *Calamaria* and outgroup taxa used in this study.

No.	Species	Specimen voucher	Locality	cyt *b*	Sources
1	*C. similis* sp. nov.	KFBG 14507	Longzhou, Chongzuo, Guangxi, China	MH445957	Yang and Zheng 2018
2	*C. similis* sp. nov.	SYS r001816	Yangchun, Yangjiang, Guangdong, China	PZ158843	This study
3	*C. similis* sp. nov.	SYS r001725	Nonggang National NR, Guangxi, China	PZ158841	This study
4	*C. similis* sp. nov.	SYS r001726	Nonggang National NR, Guangxi, China	PZ158842	This study
5	*C. alcalai*	PNM 9873	Sitio Palbong, Barangay Batong Buhay, Sablayan, Mindoro, Philippines	MT819383	Weinell et al. 2021
6	*C. andersoni*	SYS r001699	Yingjiang, Yunnan, China	MH445955	Yang and Zheng 2018
7	*C. andersoni*	HS R20101	Dehong, Yunnan, China	OQ354844	Cai et al. 2023
8	*C. andersoni*	HS R20181	Tengchong, Yunnan, China	OQ354845	Cai et al. 2023
9	*C. annamensis*	VRTC NAP-17841	Bac Huong Hoa NR, Quang Tri, Vietnam	PX607279	Korolev et al. 2026
10	*C. annamensis*	VRTC NAP-17842	Bac Huong Hoa NR, Quang Tri, Vietnam	PX607280	Korolev et al. 2026
11	*C. annamensis*	VRTC NAP-17843	Bac Huong Hoa NR, Quang Tri, Vietnam	PX607281	Korolev et al. 2026
12	*C. arcana*	KFBG 14611	Mt. Dadongshan, Guangdong, China	ON482335	Yeung et al. 2022
13	*C. arcana*	HS 17082	Mt. Dawu, Guangdong, China	OQ354835	Cai et al. 2023
14	*C. arcana*	GP 9975	Yongxing, Hunan, China	OP980549	Cai et al. 2023
15	*C. arcana*	DL R199	Mt. Wuyi, Fujian, China	OQ354834	Cai et al. 2023
16	*C. berezowskii*	GXNU DLR194	Mt. Gongga, Sichuan, China	PP747047	Liang et al. 2024
17	*C. berezowskii*	GXNU DLR195	Mt. Gongga, Sichuan, China	PP747048	Liang et al. 2024
18	*C. berezowskii*	GXNU 20221215002	Mt. Gongga, Sichuan, China	PP747049	Liang et al. 2024
19	*C. gervaisii*	KU 324661	Puguis, La Trinidad, Benguet, Luzon, Philippines	MT819384	Weinell et al. 2021
20	*C. gervaisii*	KU 334485	Narvacan, Ilocos Sur, Luzon, Philippines	MT819385	Weinell et al. 2021
21	*C. jinggangensis*	CIB-DL 20200725	Mt. Jinggangshan, Jiangxi, China	OQ354830	Cai et al. 2023
22	*C. jinggangensis*	DL 20200625-2	Mt. Jinggangshan, Jiangxi, China	OQ354831	Cai et al. 2023
23	*C. jinggangensis*	DL 20200625-3	Mt. Jinggangshan, Jiangxi, China	OQ354832	Cai et al. 2023
24	*C. jinggangensis*	DL 20200625-4	Mt. Jinggangshan, Jiangxi, China	OQ354833	Cai et al. 2023
25	*C. lumbricoidea*	KU 315159	Pasonanca NP, Zamboanga, Philippines	MT819388	Weinell et al. 2021
26	*C. lumbricoidea*	KU 334479	Mt. Lumot, Gingoog, Misamis, Philippines	MT819389	Weinell et al. 2021
27	*C. cf. lumbricoidea*	USMHC 1560	Air Itam Dam, Penang, Malaysia	MN338526	Quah et al. 2020
28	*C. mizoramensis*	MZMU 3141	Reiek, Mizoram, India	PX557820	Lalremsanga et al. 2026
29	*C. mizoramensis*	MZMU 3143	Reiek, Mizoram, India	PX557819	Lalremsanga et al. 2026
30	*C. mizoramensis*	MZMU 3144	Reiek, Mizoram, India	PX557818	Lalremsanga et al. 2026
31	*C. muelleri*	TNHC 58955	Gowa, South Sulawesi, Indonesia	MT819390	Weinell et al. 2021
32	*C. muelleri*	RMB 1283	Gowa, South Sulawesi, Indonesia	MT819391	Weinell et al. 2021
33	*C. nebulosa*	FMNH 258666	Phongsaly, Laos	MN338524	Quah et al. 2020
34	*C. palavanensis*	KU 309445	Barangay Irawan, Puerto Princessa, Palawan, Philippines	MT819386	Weinell et al. 2021
35	*C. palavanensis*	KU 311411	Mt. Mantalingahan, Rizal, Palawan, Philippines	MT819387	Weinell et al. 2021
36	*C. schlegeli*	LSUHC 10278	Bukit Larut, Perak, Malaysia	MN338525	Quah et al. 2020
37	*C. septentrionalis*	FTB 2839	unknown locality	KR814699	Pyron unpublished data
38	*C. septentrionalis*	KFBG 14506	Hainan, China	MH445956	Yang and Zheng 2018
39	*C. septentrionalis*	HS 11119 (CHS 116)	Tunxi, Huangshan, Anhui, China	MK201273	Li et al. 2020
40	*C. septentrionalis*	HS 12055 (CHS 118)	Huangshan, Anhui, China	MK201274	Li et al. 2020
41	*C. septentrionalis*	RE 30 (CHS 302)	Mangshan, Hunan, China	MK201384	Li et al. 2020
42	*C. septentrionalis*	SYS r000932 (CHS 613)	Mt. Tianjing, Guangdong, China	MK201434	Li et al. 2020
43	*C. septentrionalis*	HS R19100	Mt. Huangshan, Anhui, China	OQ354842	Cai et al. 2023
44	*C. septentrionalis*	HS 11145	Mt. Nanling, Guangdong, China	OQ354840	Cai et al. 2023
45	*C. septentrionalis*	DL 2021610-1	Huangsha, Guangxi, China	OQ354838	Cai et al. 2023
46	*C. cf. septentrionalis*	ROM 35605	Phia Oac-Phia Den NP, Cao Bang, Vietnam	AF471081	Lawson et al. 2005
47	*C. cf. septentrionalis*	ROM 35597	Phia Oac-Phia Den NP, Cao Bang, Vietnam	KX694890	Alencar et al. 2016
48	*C. synergis*	AHNU ZR24046	Mt. Jinuo, Xishuangbanna, Yunnan, China	PV745121	Zhang et al. 2025a
49	*C. yunnanensis*	ROM 41547	Simao, Yunnan, China	KX694891	Zaher et al. 2009
50	*C. yunnanensis*	YPX 503	Yunnan, China	JQ598922	Grazziotin et al. 2012
**Outgroups**					
51	*Orientocoluber spinalis*	MVZ 211019	Ningxia, China	AY486924	Nagy et al. 2004
52	*Elaphe quatuorlineata*	LSUMZ 40626	Hungary	AY486931	Nagy et al. 2004
53	*Lycodon rufozonatus*	LSUMZ 44977	China	AF471063	Lawson et al. 2005

### Phylogenetic relationships

**Figure 3.**
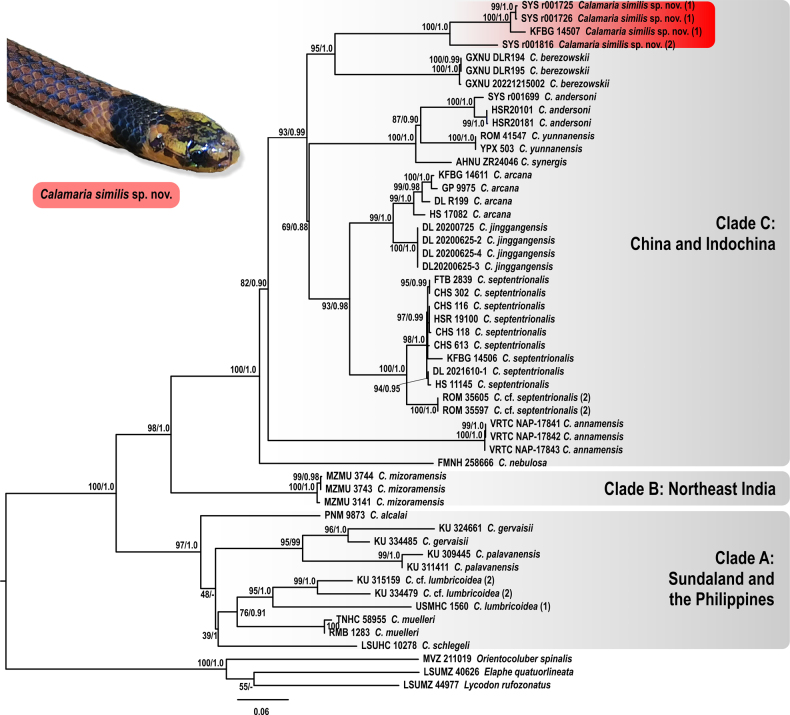
Maximum Likelihood (ML) phylogenetic tree of *Calamaria* derived from the analysis of 1,109 bp of cyt *b* mitochondrial gene fragment. For voucher specimen information and GenBank accession numbers see Table 2. Values at the nodes correspond to MLUFBS/BIPP support values. Thumbnail shows *Calamaria similis* sp. nov. in life; photograph by JJL.

ML and BI analyses yielded predominantly congruent topologies with discrepancies confined to deeper nodes within Clade A (Sundaland species), which are not pertinent to the current investigation (Fig. 3). The genus *Calamaria* was robustly confirmed as monophyletic (100/1.0; node support values subsequently shown as MLUFBS/BIPP), with all sampled species grouped into three strongly supported and geographically bounded clades.

Clade A (97/1.0) includes species from Sundaland, the Philippines, and Sulawesi and is phylogenetically recovered as sister to Clades B and C from mainland Asia and adjacent islands. Relationships within Clade A are insufficiently resolved: *Calamaria alcalai* Weinell, Leviton & Brown, 2020 was identified as sister to the rest of the clade without node support (48/-); *C. gervaisii* Duméril, Bibron & Duméril, 1854 and *C. palavanensis* Inger & Marx, 1965 exhibited a strongly supported sister species relationship (95/0.99); *C. muelleri* Boulenger, 1896 was moderately supported as a sister species to *C. lumbricoidea* Boie, 1827 (76/0.91), while the phylogenetic position of *C. schlegeli* Duméril, Bibron & Duméril, 1854 remains essentially unresolved. A significant divergence was recovered between the specimen USMHC 1560 of *C. lumbricoidea* from the Penang State, Malaysia, and the two Philippine samples of *C. cf. lumbricoidea* (95/1.0) (Fig. 3).

Clade B comprises three specimens of the recently described *C. mizoramensis* from Mizoram State, India (100/1.0), establishing a profoundly different mtDNA-lineage sister to Clade C (Fig. 3).

Clade C (100/1.0) comprises species from Indochina and East Asia. *Calamaria nebulosa* Lee, 2021 is the sister taxon to the other members of this clade, but with low node support (82/0.90) (Fig. 3). The recently revalidated *C. annamensis* was reconstructed as a sister lineage to all the remaining species, but with low node support (82/0.90). Sister species relationships were identified between *C. arcana* and *C. jinggangensis* Cai, Jiang, Wu, Huang, Fei & Ding, 2023 (99/1.0), which form a moderately supported (93/0.98) sister clade to the *C. septentrionalis* complex (100/1.0). Within the latter, a significant divergence was recovered between *C. cf. septentrionalis* Boulenger, 1890 from Cao Bang Province, Vietnam (ROM 35605, ROM 35597; 100/1.0), and Chinese populations of true *C. septentrionalis* (98/1.0). Three species from Yunnan (*C. yunnanensis* Chernov, 1962, *C. andersoni* Yang & Zheng, 2018, and *C. synergis*) constituted a distinct subclade (100/1.0) with *C. yunnanensis* recovered sister to *C. andersoni* (87/0.90), and *C. synergis* recovered sister to that species pair.

The newly collected specimens of *Calamaria* sp. from Guangdong and Guangxi formed a strongly supported clade (95/1.0) sister to *C. berezowskii* from Sichuan Province, China (Fig. 3). Monophyly of *Calamaria* sp. from Guangdong and Guangxi was strongly supported (100/1.0), with the specimen SYS r001816 from Guangdong forming a distinct sister lineage (lineage 2, Guangdong) to the remaining three specimens from Guangxi (lineage 1, Guangxi) (Fig. 3).

### Genetic distances

Table 3 shows the uncorrected *p*-distances for the cyt *b* gene fragment among the examined *Calamaria* species. Pairwise interspecific distances varied from 6.29% (between *C. jinggangensis* and *C. arcana*) to 24.95% (between *C. berezowskii* and *C. schlegeli*). The Guangxi-Guangdong populations of *Calamaria* sp. exhibited a minimal divergence of 14.97% from *C. nebulosa* and 23.80% from *C. cf. lumbricoidea*. Intraspecific divergence among the Guangxi (lineage 1) and Guangdong (lineage 2) populations of *Calamaria* sp. was high and comprised *p* = 10.78%, while the divergence among the Guangxi specimens was minor at *p* = 0.8%. Significant intraspecific divergence was also observed in *C. gervaisii* (8.87%) and *C. cf. lumbricoidea* (7.48%). This level of intraspecific divergence is comparable to or slightly higher than that reported in other *Calamaria* species, such as *C. gervaisii* and *C. cf. lumbricoidea*, but it also approaches the lower range of interspecific divergences observed among some recognized *Calamaria* species.

**Table 3.** Uncorrected *p*-distances (percentage) between *Calamaria* species based on 1,109 base pairs from the mitochondrial cyt *b* gene. Notes: n/a = Not available.

Taxon		1	2	3	4	5	6	7	8	9	10	11	12	13	14	15	16	17	18	19	20
1	*C. similis* sp. nov. 1	**0.80**																			
2	*C. similis* sp. nov. 2	10.78	**n/a**																		
3	*C. alcalai*	23.17	21.04	**n/a**																	
4	*C. andersoni*	20.06	17.86	20.73	**1.20**																
5	*C. annamensis*	19.26	18.46	20.73	17.86	**0.20**															
6	*C. arcana*	18.04	19.16	20.27	18.34	17.34	**2.94**														
7	*C. berezowskii*	17.56	17.76	23.68	18.96	17.66	17.71	**0.20**													
8	*C. gervaisii*	21.27	19.65	15.38	19.91	20.72	18.75	22.33	**8.87**												
9	*C. jinggangensis*	17.07	17.66	19.21	16.17	18.16	6.29	18.46	15.87	**0.00**											
10	*C. lumbricoidea* 1	22.26	20.36	18.29	21.96	21.46	20.96	22.06	19.05	20.66	**n/a**										
11	*C. cf. lumbricoidea* 2	23.80	22.13	15.57	22.43	21.66	20.41	22.85	17.49	18.92	15.57	**7.48**									
12	*C. mizoramensis*	19.76	20.96	15.85	19.26	20.86	16.84	23.25	15.87	15.27	18.86	19.69	**0.00**								
13	*C. muelleri*	22.50	22.40	14.90	21.46	20.53	17.81	21.05	15.09	16.49	15.86	13.32	17.42	**0.63**							
14	*C. nebulosa*	18.86	14.97	18.29	15.57	16.67	17.37	15.67	19.04	17.07	18.56	20.00	20.96	18.98	**n/a**						
15	*C. palavanensis*	20.70	18.77	14.64	19.60	19.39	20.42	22.03	13.09	17.48	16.09	15.84	17.17	15.94	18.28	**2.38**					
16	*C. schlegeli*	22.26	20.66	16.77	22.65	20.66	20.51	24.95	16.02	19.76	19.16	17.24	18.26	16.18	17.66	15.91	**n/a**				
17	*C. septentrionalis* 1	16.30	17.50	21.07	14.01	17.40	11.35	18.00	18.95	9.85	21.22	20.88	17.86	20.32	18.33	18.24	22.55	**0.50**			
18	*C. cf. septentrionalis* 2	17.66	17.96	22.56	14.77	17.27	10.18	16.37	19.66	10.78	20.66	20.93	19.76	21.15	16.77	19.06	22.16	4.03	**0.00**		
19	*C. synergis*	17.96	15.87	18.90	8.08	16.97	14.37	18.06	19.20	12.28	20.36	19.22	17.66	19.29	14.37	18.60	19.46	12.71	12.87	**n/a**	
20	*C. yunnanensis*	20.06	19.46	21.95	11.38	19.66	18.19	18.66	22.38	15.87	20.96	20.45	21.56	23.33	15.87	21.79	23.35	15.34	14.37	7.78	**0.00**

### Taxonomic conclusion

Based on the combined evidence from detailed morphological comparisons and mitochondrial DNA analyses, we consider the *Calamaria* populations from Guangdong Province and Guangxi ZAR, China, previously referred to as *Calamaria pavimentata*, to represent a distinct evolutionary lineage. Although mitochondrial DNA data from topotypic *C. pavimentata* from Java Island, Indonesia, are currently unavailable, the observed differences in ventral and subcaudal scale counts, body proportions, and the pronounced geographic disjunction between Java and southern China support the recognition of this population as a distinct species, which we formally describe below:

#### Calamaria similis sp. nov.

Taxon classificationAnimaliaSquamataColubridae

35E889A3-0718-542E-95AE-1918E9FEC1C0

https://zoobank.org/059CB9F9-4DF6-4FC4-966F-2408B508C12E

**Figure 4.**
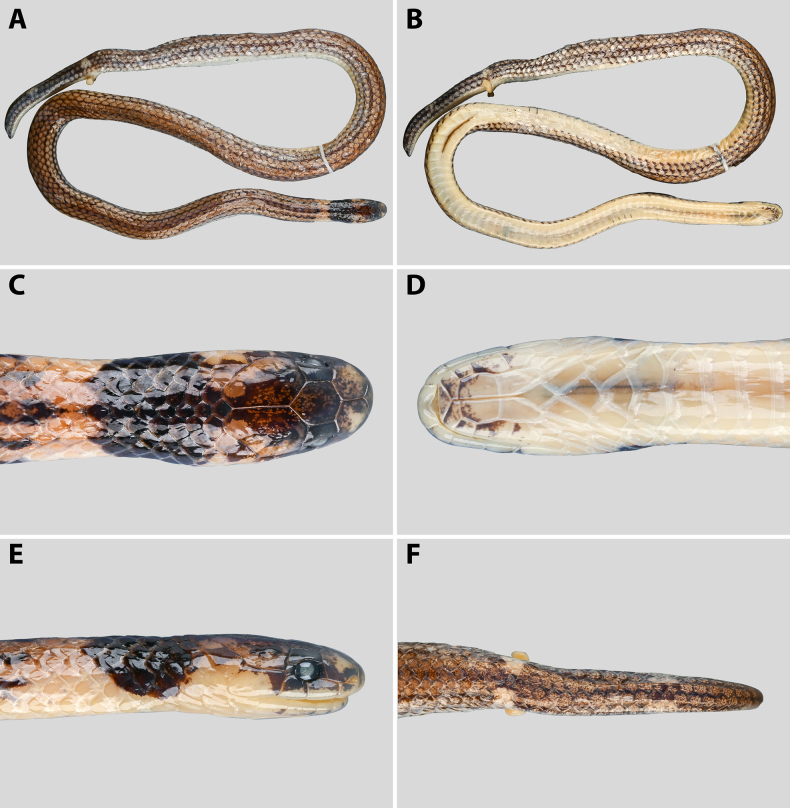
*Calamaria similis* sp. nov, adult male (SYS r001816, holotype) in preserved. **A**. Dorsal view of body; **B**. Ventral view of body; **C**. Dorsal view of head; **D**. Ventral view of head; **E**. Lateral view of head, right side; **F**. Dorsal view of tail (posterior body). Photographs by SQ.

**Figure 5.**
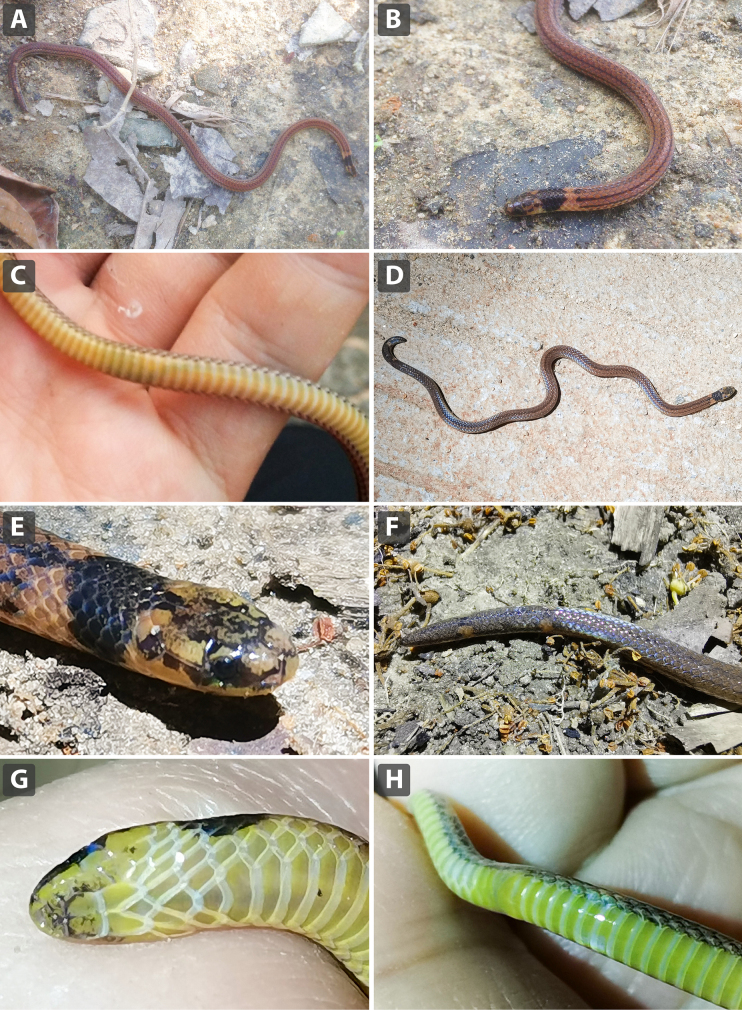
*Calamaria similis* sp. nov, in life, from Yangchun, Yangjiang, Guangdong, China. **A–C**. Adult male (SYS r001816, holotype); **A**. Dorsal view of body; **B**. Anterior view of body; **C**. Ventral view of body; **D–H**. Not collected, sex undetermined; **D**. Dorsal view of body; **E**. Anterolateral view of body; **F**. Dorsal view of tail; **G**. Ventral view of head; **H**. Ventral view of body (cloacal region). Photographs by JJL.

**Figure 6.**
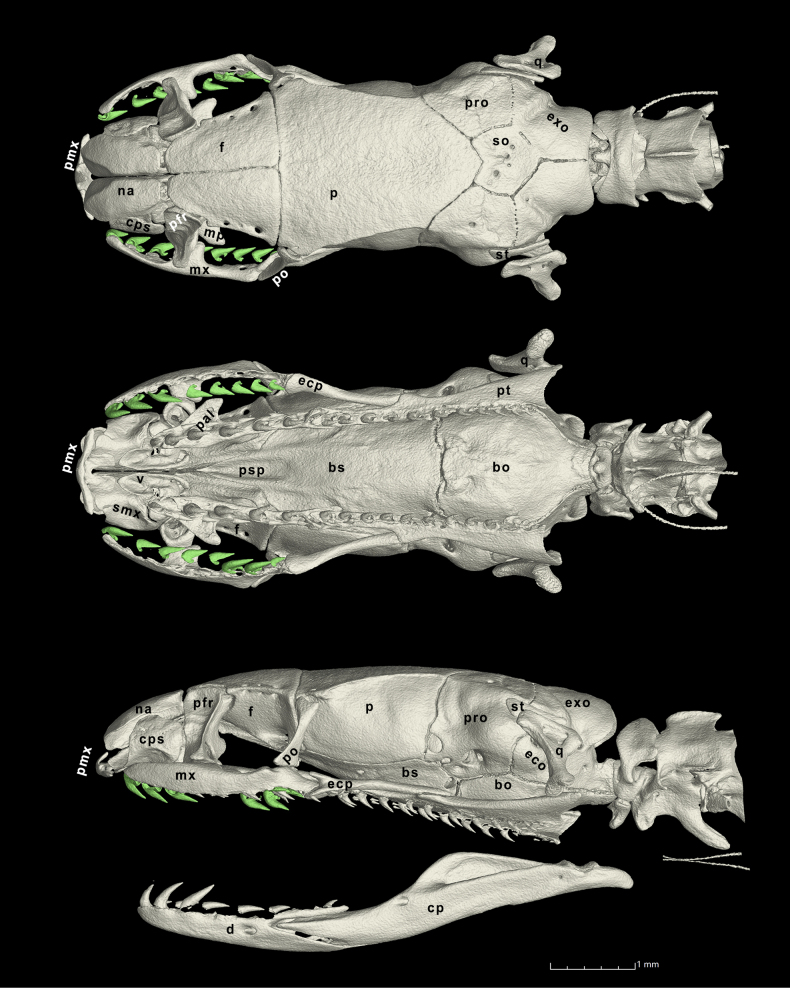
Three-dimensional model of the skull of *Calamaria similis* sp. nov (SYS r001816, holotype). Top, dorsal view; middle, ventral view; bottom, lateral view. Abbreviations: bo = basioccipital, bs = basisphenoid, cps = conchal process of septomaxilla, ecp = ectopterygoid, exo = exoccipital, mp = maxillary process of palatine, mx = maxilla, na = nasal, p = parietal, pal = palatine, pfr = prefrontal, pmx = premaxilla, po = postorbital, pro = preoccipital, psp = parasphenoid rostrum, pt = pterygoid, q = quadrate, smx = septomaxilla, so = supraoccipital, st = supratemporal. Implemented by JSS.

**Figure 7.**
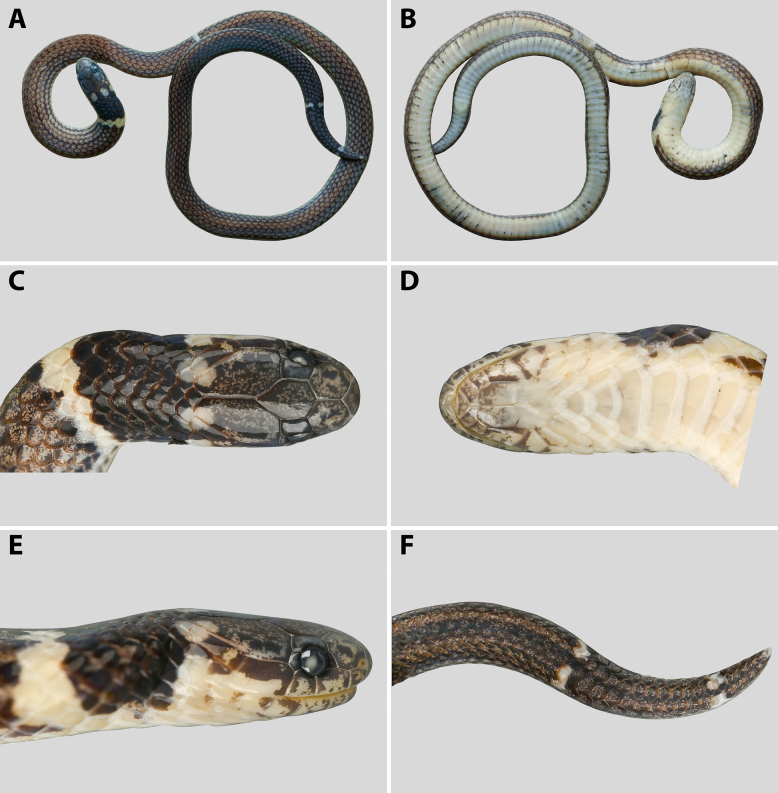
*Calamaria similis* sp. nov, subadult female (SYS r001725, paratype) in preserved. **A**. Dorsal view of body; **B**. Ventral view of body; **C**. Dorsal view of head; **D**. Ventral view of head; **E**. Lateral view of head, right side; **F**. Dorsal view of tail (posterior body). Photographs by SQ.

**Figure 8.**
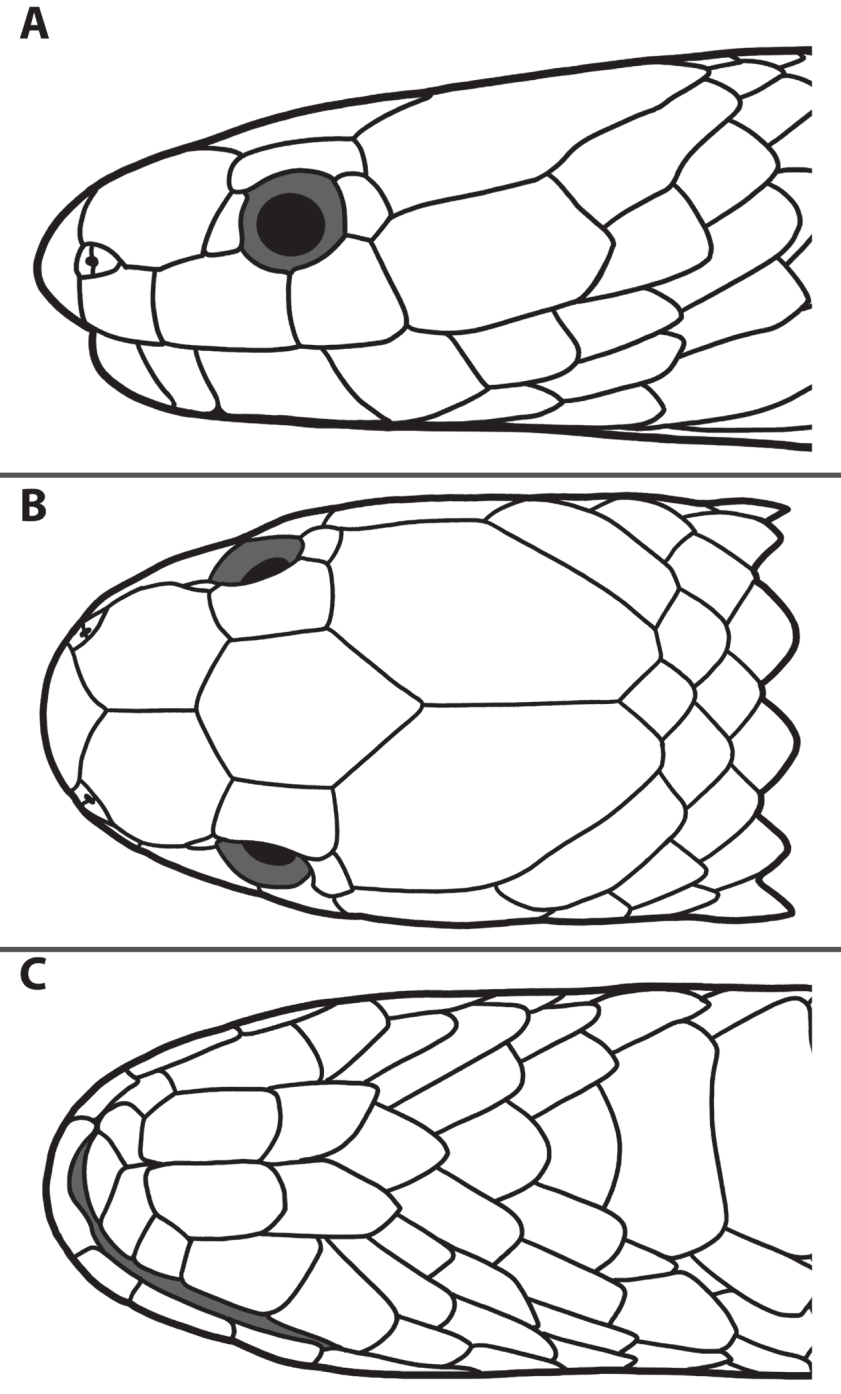
Head scalation of a subadult female *Calamaria similis* sp. nov (SYS r001725, paratype). **A**. Lateral view of the head, right side; **B**. Dorsal view of the head; **C**. Ventral view of the head. Line drawings by AMK, not to scale.

**Figure 9.**
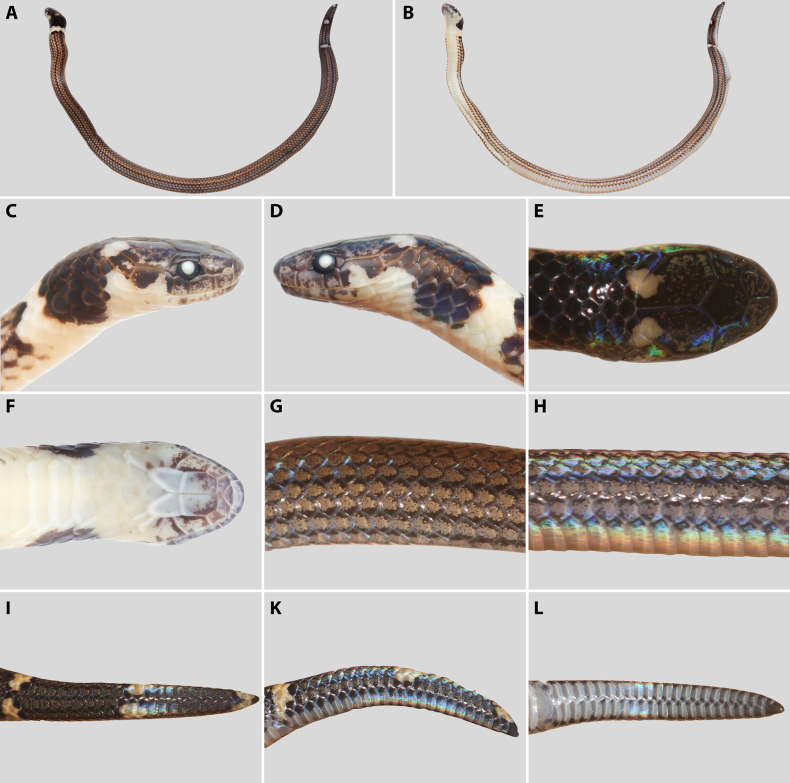
*Calamaria similis* sp. nov., subadult male (KFBG 14507, paratype) in preserved. **A**. Dorsal view of body; **B**. Ventral view of body; **C**. Lateral view of head, right side; **D**. Lateral view of head, left side; **E**. Dorsal view of head; **F**. Ventral view of head; **G**. Dorsal view of midbody; **H**. Dorsolateral view of midbody; **I**. Dorsal view of tail; **K**. Lateral view of tail; **L**. Ventral view of tail. Photographs by JHY.

Tables 4, 5; Figs 4, 5, 6, 7, 8, 9

##### Chresonymy.

*Calamaria pavimentata* – Yang and Zheng (2018, in part); Lee (2021, in part); Yeung et al. (2022; in part); Cai et al. (2023: in part); Poyarkov et al. (2023: in part); Liang et al. (2024, in part); Zhang et al. (2025a, 2025b; in part); Lalremsanga et al. (2026, in part).

*Calamaria pavimentata pavimentata* – David et al. (2023: in part).

##### Type material.

***Holotype***: China • 1 ♂; Guangdong Province, Yangjiang City, Yangchun City, Shuangjiao Town, Qifendong Village [now Dahe Village]; 22.07061848°N, 111.41304597°E; altitude 44 m a.sl.; collected by JJL on 12 August 2017; SYS r001816. ***Paratypes***: China • 1 subadult ♀; Guangxi ZAR, Chongzuo City, Longzhou County, Nonggang National Nature Reserve; 22.46164593°N, 106.97141398°E; altitude 350 m a.s.l.; collected by CZR on 24 May 2017; SYS r001725 • 1 subadult ♂; Guangxi ZAR, Chongzuo City, Longzhou County, Nonggang National Nature Reserve; 22.46164593°N, 106.97141398°E; altitude 350 m a.s.l.; collected by CZR on 24 May 2017; SYS r001726 • 1 subadult ♂; Guangxi ZAR, Chongzuo City, Longzhou County, Shuikou Town, Shangjin Village; 22.317845°N, 107.022622°E; altitude ca. 300 m a.s.l.; collected by JHY in 2013; KFBG 14507.

##### Diagnosis.

*Calamaria similis* sp. nov. is distinguished from all other congeners by the combination of the following morphological characters: Maxillary teeth eight, unmodified; rostral higher than wide; prefrontal shorter than frontal and contacting the first two supralabials; mental not in contact with anterior chin shields; dorsal scales in 13–13–13 rows, smooth throughout; a single preocular and postocular; four supralabials, with the 2^nd^ and 3^rd^ contacting the eye; five infralabials; six scales surrounding the paraparietal; 145–168 ventrals (145–155 in males, 168 in female); 15–23 paired subcaudals (20–23 in males, 15 in female); a relatively short tail (6.4–9.3% of total length in males, 6.1% in the female), thick and nearly cylindrical, gradually tapering to an obtuse point. Dorsal coloration dark brown to blackish brown, the body marked with five longitudinal rows of dark-edged scales, forming indistinct, discontinuous stripes of variable clarity along the body; two nuchal collars are present, the first is a larger, dark brown collar, and the second is smaller and without pattern; two pairs of light spots are present on the dorsal tail, variably developed and sometimes indistinct. The ventral surface yellowish white, immaculate; underside of tail lacking a broad median black stripe extending posteriorly to the tail tip.

##### Description of the holotype, SYS r001816, adult male (see Figs 4, 5A–C).

Specimen in good condition, slightly discolored due to preservation after approximately nine years in preservative fluid (Fig. 4). Body slender, cylindrical (SVL 170.0 mm); tail short, thinner than body, thick and nearly cylindrical, gradually tapering to an obtuse point (TaL 17.4 mm, TaL/TL 9.3%). Head small, elliptical in dorsal view (head length 6.1 mm; head width 3.5 mm; head height 2.4 mm). Eye small, round (eye diameter 0.8 mm), smaller than eye-nostril distance 1.1 mm but larger than eye-mouth distance 0.5 mm.

***Head scalation***. Rostral broader than high (rostral width 1.2 mm, rostral height 0.9 mm); dorsal portion visible from above approximately half the length of the prefrontal suture. Prefrontal shorter than frontal, not entering orbit, in contact with the first and second supralabials. Frontal hexagonal, longer than wide (frontal length 1.8 mm, frontal width 1.4 mm). Temporals absent. Paraparietal surrounded by six scales. One preocular on each side, higher than wide, slightly taller than postocular but shorter than eye diameter. One postocular on each side, also higher than wide. Nasals divided, small, bordered by rostral, prefrontal, and 1^st^ supralabial. Four supralabials on each side, the 2^nd^ and 3^rd^ entering the orbit; the 4^th^ largest in height (relative size: 4 > 2 > 3 > 1), but the 2^nd^ one, rather low, is as long as the 4^th^. Five infralabials on each side, the first three pairs contacting anterior chin shields; the 1^st^ pair meeting at the midline; the 4^th^ largest. Anterior chin shields much longer than wide, slightly pentagonal, meet completely along their longest sides. Posterior chin shields are shorter, in contact anteriorly, and separated by the preventral at approximately one-third of their length.

***Body scalation***. Dorsal scale rows 13–13–13 throughout body, all smooth. Ventrals 150 (+ 1 preventral); subcaudals 21, all paired; dorsocaudal reduction from 9 to 8 occurring posterior to subcaudal 3, from 8 to 6 occurring posterior to subcaudal 4, and from 6 to 5 occurring posterior to subcaudal 15; Cloacal plate undivided.

***Coloration of the holotype***. In life (Fig. 5A–C), the dorsal surface of head is reddish brown, with blackish pigment forming a cap-like marking dorsally; the lateral surfaces of the head are slightly paler than the dorsum, with blackish pigment forming a wavy longitudinal mask extending from the rostral region to the nape. The ventral surface of the head is pale yellow, with irregular dark brown markings restricted to the mental, the infralabials, and anterior chin-shields. The dorsal surface of the body is reddish brown. Two nuchal collars are present, the first is a distinct dark brown collar, slightly shorter than the head length and extending laterally onto the sides of the neck; the second is smaller, without pattern, and approximately one to two dorsal scales in length. Five longitudinal series of interrupted dark brown stripes are visible along the body, extending from behind the posteriormost collar to the tip of the tail. The margins of the dorsal scales are heavily suffused with black pigment, forming an almost reticulate pattern. The ventrals are pale yellow, with dark brown blotches along the outermost lateral margins, forming a dashed line parallel to the longitudinal stripes. The tail dorsum is slightly darker than the body dorsum, and the longitudinal stripes fade posteriorly. Two pairs of small, faint yellowish-brown spots are present on the dorsal tail, one pair at the base and one posteriorly. Subcaudal surface pale yellow, generally immaculate.

In preservative (Fig. 4), the dorsal surface of the head, body, and tail fade to brown. Two nuchal collars remain in strong contrast. The dark brown longitudinal stripes become much more diffuse, whereas the reticulate pattern formed by the heavily pigmented margins of the dorsal scales become more conspicuous. The pale spots on the tail turn light yellowish brown. The ventral surfaces of the head, body, and tail are uniformly pale yellowish white.

***Skull morphology of holotype***. (Fig. 6) The skull is subcylindrical in general shape. In dorsal view, the anterior margin of the premaxilla is flat, the nasals and septomaxillae are relatively strong and laterally expanded, the conchal processes of the septomaxillae are broad, flattened, and well-developed. The frontals are approximately right-triangle-shaped with their anterior tip rounded. The parietal is scutiform, longer than wide, while the supraoccipital is subpentagonal. The prootic and exoccipitals are both well developed and similar in size, forming the widest part of the braincase.

In ventral view, the vomers are well-developed with their anterior tips nearly reach the vomerine processes of premaxilla. The maxillary processes of palatines are triangular and laterally expanded while the choanal processes degenerated, almost invisible. The pterygoid is slender and lanceolate in shape, twice the length of the palatine. The basisphenoid and basioccipital lack a ventral process. The parasphenoid rostrum of the basisphenoid is sharp and triangular shaped. The basioccipital is relatively flat and laterally expanded.

In lateral view, the ascending process of premaxilla is slender and greatly reduced. The conchal process of the septomaxilla is anteroposteriorly expanded, reaching the lateral edge of nasals. The prefrontal and postorbital are similar in height, the bottom tip of the postorbital reaches the top margin of the maxilla. The ectopterygoid is biforked, the labial furcula of ectopterygoid is oval, and distinct from the lingual process. The lingual process projects anterodorsally, overlaps the dorsal margin ectopterygoid process of the maxilla, visible in lateral view but not visible in ventral view.

The mandible is slender and moderately curved. The prearticular crest of the compound bone is prominent while the surangular crest is absent.

***Dentition of the holotype***. (Fig. 6) The maxillary teeth are conical, laterally compressed, and posteriorly recurved at the tip, consistent with the description of unmodified maxillary teeth in Inger and Marx (1965). The morphology of palatine teeth, pterygoid teeth, and dentary teeth show no significant specialization relative to the maxillary teeth. Maxillary teeth 8, palatine teeth 5, pterygoid teeth 11, dentary teeth 7 on each side.

##### Variation.

(Figs 7, 8, 9) Variation in measurements and scalation of the type series is summarized in Table 4. Sexual dimorphism is evident in several characters. The female possesses significantly more ventral scales than males (VEN 168 vs 145–155), but fewer subcaudal scales (SC 15 in female vs 21–23 in males). Tail proportions also differ markedly between sexes: males have relatively longer tails (TaL/TL ratio 6.4–9.3%), whereas the female exhibits a shorter relative tail length (TaL/TL ratio 6.1%). Coloration shows moderate intraspecific variation. The dorsal ground color varies from reddish brown to dark brown, with five dark longitudinal stripes running along the body. Supralabials and infralabials are cream to yellowish white, upper portions of supralabials occasionally with scattered dark pigmentation. Ventral coloration is uniform, ranging from yellowish white to pale yellow, without markings. The subcaudal region is similar in color to the ventrals or slightly darker and lacks a dark longitudinal stripe on the underside of the tail. The tail tip varies in pigmentation, being uniformly dark, distinctly darker than the remainder of the tail, or only weakly pigmented.

**Table 4.** Selected measurements and meristic characters of *Calamaria similis* sp. nov. and *Calamaria pavimentata*. Notes: H = holotype; P = paratype; M = adult male; F = adult female; SM = subadult male; SF = subadult female; * = damaged specimen; n/a = not available.

Voucher	Locality	Sex	SVL (mm)	TaL (mm)	ASR-MSR-PSR	VEN	Anal	SC	SL	SL-E	IL	PrO	PoO	Source
***Calamaria similis* sp. nov**.														
SYS r001816^H^	Yangchun, Yangjiang, Guangdong, China	M	170.0	17.4	13-13-13	150	1	21	4/4	2-3/2-3	5/5	1/1	1/1	3
KFBG 14507^P^	Shangjin, Longzhou, Guangxi, China	SM	95.3	9.7	13-13-13	155	1	23	4/4	2-3/2-3	5/5	1/1	1/1	3
SYS r001726^P^	Nonggang National NR, Guangxi, China	SM	142.9	9.8	13-13-13	145	1	20	4/4	2-3/2-3	5/5	1/1	1/1	3
SYS r001725^P^	Nonggang National NR, Guangxi, China	SF	144.5	9.3	13-13-13	168	1	15	4/4	2-3/2-3	5/5	1/1	1/1	3
***Calamaria pavimentata***														
MNHN-RA-0.3298^H^	Java, Indonesia	M	182.1	24.0	13-13-13	151	1	27	4/4	2-3/2-3	5/5	1/1	1/1	3
MNHN-RA-0.3297^*^	Java, Indonesia	SF	115.0	10.0	13-13-13	136	1	13	4/4	2-3/2-3	5/5	1/1	1/1	1
***Calamaria cf. pavimentata***														
No. 1016^*^	Jinxiu, Laibin, Guangxi, China	M	180.0	14.0	13-13-13	179	1	22	4/4	2-3/2-3	5/5	1/1	1/1	2
No. 2009^*^	Jinxiu, Laibin, Guangxi, China	F	273.0	13.0	13-13-13	n/a	n/a	n/a	4/4	2-3/2-3	5/5	1/1	1/1	2

Sources: 1 = Duméril et al. (1854); 2 = Fan (1931); 3 = this study.

##### Etymology.

The specific epithet *similis* is a Latin adjective meaning similar, referring to the strong morphological resemblance of this species to *Calamaria pavimentata*. This similarity has resulted in the species being previously misidentified and treated as *C. pavimentata* in numerous earlier studies (e.g., Yang and Zheng 2018). We propose the following common names for the new species: “Similar Reed Snake” (English), “拟尖尾两头蛇” (nĬ jiān wěi liăng tóu shé, Chinese), “Rắn mai gầm tương đồng” (Vietnamese), and “Сходная карликовая змея” (Skhodnaya karlikovaya zmeya, Russian).

##### Comparison.

Comparative morphological data for the new species and currently recognized members of the genus *Calamaria* from mainland China and the Indo-Burma Region are presented in Table 5. *Calamaria similis* sp. nov. differs from all congeners, particularly those referred to the *Calamaria pavimentata* species complex, by a unique combination of meristic characters, body proportions, and coloration, as detailed below:

**Table 5.** Comparison of morphological characters of *Calamaria similis* sp. nov. with those of congeners occurring in China and the Indo-Burma region. Symbols: (1) = Supralabials; (2) = Supralabials contacting orbit; (3) = Preocular (1 = present, 0 = absent); (4) = Mental touching chin shields (1 = yes, 0 = no); (5) = Number of scales contacting paraparietal; (6) = VEN in males; (7) = VEN in females; (8) = SC in males; (9) = SC (females); (10) = maximum TL (mm) in males; (11) = maximum TL (mm) in females; (12) = ratio TaL/TL (%) in males; (13) = ratio TaL/TL (%) in females; (14) = Tail: tapering (2), slightly tapered (1), or not (0); (15) = End of tail; (16) = Dorsal color; (17) = Ventrals color. Notes: N/a: Not available.

Species	(1)	(2)	(3)	(4)	(5)	(6)	(7)	(8)	(9)	(10)	(11)	(12)	(13)	(14)	(15)	(16)	(17)	Sources
*C. similis* sp. nov.	4	2/3	1	0	6	145–155	168	20–23	15	187	154	6.4–9.3	6.1	2	obtuse point	reddish brown to dark brown with five narrow dark longitudinal stripes	pale yellow, immaculate	This study
*C. abramovi*	4	2/3	1	0	6	159	174	26	20	139	482	13.3	7.1	2	sharp point	uniform black	yellow-orange spots	Orlov 2009
*C. andersoni*	4	2/3	1	0	6	164–172	186	20–23	14	351	312	8.8–9.2	5.8	0	obtuse point	brownish with faint narrow black lateral stripes	orange-yellow, immaculate	Yang and Zheng 2018; Zhang et al. 2025b
*C. annamensis*	4	2/3	1	0	6	182	196–204	22	14–15	304	552	6.6	3.9–4.7	1	obtuse point	uniform dark brown to olive-brown	light orange to salmon, with small black spots	Korolev et al. 2026
*C. arcana*	4	2/3	1	0	6	170–176	192	20–22	12	303	n/a	7.2–11.8	4.7	0	obtuse point	brownish	orangish-red, immaculate	Yeung et al. 2022; Zhang et al. 2023
*C. berezowskii*	4	2/3	1	0	6	149–155	153–171	22–25	12–16	290	245	6.6–10.5	6.5–6.9	0	obtuse point	blackish-brown or brown	light khaki or white	Inger and Marx 1965; Liang et al. 2024
*C. buchi*	4	2/3	1	1	5	n/a	221–236	n/a	13–14	n/a	466	n/a	3.9–4.1	1	obtuse point	blackish with small light spots	yellow, immaculate	Inger and Marx 1965; Nguyen et al. 2009
*C. concolor*	5	2/3	1	1	5	209	n/a	19	n/a	578	n/a	7.2	n/a	1	obtuse point	uniform orangish-red	bright red, immaculate	Orlov et al. 2010; Nguyen et al. 2025
*C. dominici*	4 or 5	2/3 or 3/4	1	1 or 0	6	n/a	174	n/a	17 or 18	n/a	421	n/a	6.2	1	obtuse point	dark with irregular yellow blotches	dark with few yellow blotches & bands	Ziegler et al. 2019
*C. gialaiensis*	4	2/3	1	1	5	191	n/a	23	n/a	457	n/a	8.1	n/a	0	rounded	light greyish-brown with a faint dark neck collar and a few dark blotches along the posterior vertebral region	yellowish beige, immaculate	Nguyen et al. 2009
*C. jinggangensis*	4	2/3	1	0	6	157–158	179	20	12	260	364	15.0	3.6	0	obtuse point	brownish black	dark orange	Cai et al. 2023; Zhang et al. 2024
*C. lovii ingermarxorum*	4	2/3	0	1	6	205	n/a	23	n/a	318	n/a	7.4	n/a	0	blunt	immaculate bluish-grey with light spots on four lateral neck scales	dark gray, immaculate	Darevsky and Orlov 1992
*C. lumbricoidea*	5	3/4	1	1	4 or 5	144–196	137–229	17–27	13–21	498	642	6.3–11.4	3.9–8.3	1	sharp point	black with narrow cream or yellow rings; head red or pink in juveniles	yellow with black ventral scales forming bands	Inger and Marx 1965
*C. mizoramensis*	4	2/3	1	0	6	147–155	166–175	22–27	13–15	251	258	10.3–13.1	4.7–6.5	0	obtuse point	dark brown to blackish-brown with three to six narrow longitudinal stripes	yellow, immaculate	Lalremsanga et al. 2026
*C. nebulosa*	4	2/3	0	0	6	n/a	179	n/a	22	n/a	354	n/a	7.9	0	obtuse point	bluish-grey	yellow, immaculate	Lee 2021
*C. pavimentata*	4	2/3	1	0	6	151	136	27	13	206	125	11.6	8.0	2	obtuse point	blackish-brown with narrow dark longitudinal stripes	yellowish white, immaculate	Duméril et al. 1854; This study
*C. sangi*	4	2/3	1	1	5 or 6	190	n/a	19	n/a	373	n/a	6.2	n/a	1	obtuse point	greyish-brown with fine dark mottling	cream with narrow dark transverse bands	Nguyen et al. 2009
*C. schlegeli*	5	3/4	1 or 0	0	5 or 6	129–161	136–180	25–44	19–37	391	395	11.1–21.3	7.3–14.4	0	blunt	immaculate grey or brown; head variably pink, yellow, and/or brown	cream, immaculate	Inger and Marx 1965
*C. septentrionalis*	4	2/3	1	0	6	148–166	168–188	15–19	6–11	344	384	6.3–8.6	2.6–4.3	0	broadly rounded	dark brown or black dorsally, usually with a narrow yellow ring about six to eight scales behind the head	yellow, with small black spots	Inger and Marx 1965
*C. strigiventris*	4	2/3	1	0	6	130–168	176–183	29–31	20–22	362	367	11.2–17.9	8.4–8.6	2	abruptly to point	uniform gray-brown	bright yellow with longitudinal black stripes	Poyarkov et al. 2019
*C. synergis*	4	2/3	1	0	5	161–166	n/a	20–23	n/a	284	n/a	6.6–9.2	n/a	0	obtuse point	blackish-brown	light khaki, immaculate	Zhang et al. 2025a
*C. thanhi*	4	2/3	0	0	6 or 7	184	198	28	21	461	455	9.9	6.8	2	gradually to a point	dark with 4–6 light body bands	yellow, immaculate	Ziegler et al. 2005, 2007
*C. yunnanensis*	4	2/3	0	0	6	167–184	199	15–20	19	300	516	8.2–8.8	5.4–8.2	2	obtuse point	blue-grey to olive-brown	orange-yellow or bright red, immaculate	Lee 2021; Zhang et al. 2025b

*Calamaria similis* sp. nov. is readily distinguished from *C. lumbricoidea* (distributed in Thailand, Malaysia, Singapore, Indonesia, Brunei, and the Philippines) and *C. schlegeli* (Thailand, Malaysia, Indonesia, and Singapore) by having four supralabials, with the second and third in contact with the eye (vs five supralabials, with the third and fourth in contact). Furthermore, both species are restricted to areas south of the Isthmus of Kra in Peninsular Malaysia and have not been recorded from mainland Indochina.

*Calamaria similis* sp. nov. also differs from *C. lovii ingermarxorum* Darevsky & Orlov, 1992 (Gia Lai Province, Vietnam), *C. nebulosa* (Phongsaly Province, Laos), *C. thanhi* Ziegler & Le, 2005 (Quang Tri Province, Vietnam), and *C. yunnanensis* (Yunnan Province, China; possibly Dien Bien Province, Vietnam) by presence of a preocular scale (vs absent). It further differs from *C. buchi* Marx & Inger, 1955 (Lam Dong Province, Vietnam), *C. concolor* Orlov, Nguyen, Nguyen, Ananjeva & Ho, 2010 (Hue and Da Nang cities, Vietnam), *C. gialaiensis* Ziegler, Nguyen & Nguyen, 2008 (Gia Lai Province, Vietnam), *C. lovii ingermarxorum*, *C. lumbricoidea*, and *C. sangi* Nguyen, Koch & Ziegler, 2009 (Quang Ngai Province, Vietnam) in having the mental not contacting the chin shields (vs contacting).

*Calamaria similis* sp. nov. differs from *C. abramovi* Orlov, 2009 (Quang Ngai Province, Vietnam) by having slightly fewer ventral scales in females (VEN 168 vs 174), fewer subcaudal scales in both sexes (SC 20–23 in males and 15 in females vs 26 in males and 20 in females), a slightly shorter relative tail length in males (TaL/TL 6.4–9.3% vs 13.3%), a blackish-brown dorsal coloration with narrow dark longitudinal stripes (vs uniformly black), and presence of a nuchal collar without a pattern (vs absent).

Compared with *Calamaria andersoni* (Yunnan Province, China), *Calamaria similis* sp. nov. has lower ventral scale counts in both sexes (VEN 145–155 in males and 168 in females vs 164–172 in males and 186 in females), a smaller total length in both sexes (maximum TL 187 mm in males and 154 mm in females vs 351 mm in males and 312 mm in females).

It is distinct from *Calamaria dominici* Ziegler, Tran & Nguyen, 2019 (Lam Dong Province, Vietnam) in having lower ventral scale counts in females (168 vs 174), slightly fewer subcaudal scales in females (15 vs 17–18), a smaller total length in females (maximum TL 154 mm vs 421 mm), a blackish-brown dorsal coloration with narrow dark longitudinal stripes (vs dark with irregular yellow blotches), and presence of patternless nuchal collar (vs absent).

It differs from *Calamaria septentrionalis* (southern China and northern Vietnam) in having more subcaudal scales in both sexes (SC 22–23 in males and 15 in females vs 15–19 in males and 6–11 in females), a smaller total length in both sexes (maximum TL 187 mm in males and 154 mm in females vs 344 mm in males and 384 mm in females), a longer relative tail length in females (TaL/TL 6.1% vs 2.6–4.3%), and an obtusely pointed tail tip (vs broadly rounded).

Compared with *Calamaria strigiventris* Poyarkov, Nguyen, Orlov & Vogel, 2019 (Lam Dong and Khanh Hoa provinces, Vietnam), *Calamaria similis* sp. nov. differs by having lower ventral scale counts in females (VEN 168 vs 176–183), fewer subcaudal scales in both sexes (SC 20–23 in males and 15 in females vs 29–31 in males and 20–22 in females), a smaller total length in both sexes (maximum TL 187 mm in males and 154 mm in females vs 362 mm in males and 367 mm in females), a shorter relative tail length in both sexes (TaL/TL 6.4–9.3% in males and 6.1% in females vs 11.2–17.9% in males and 8.4–8.6% in females), presence of patternless nuchal collar (vs absent), presence of two pairs of light spots on the dorsal tail (vs absent).

From members of the *Calamaria pavimentata* species complex: *Calamaria similis* sp. nov. differs from *C. annamensis* (Quang Tri Province, Vietnam) by having lower ventral scale counts in both sexes (VEN 145–155 in males and 168 in females vs 182 in males and 196–204 in females), a smaller total length in both sexes (maximum TL 187 mm in males and 154 mm in females vs 304 mm in males and 552 mm in females), a slightly longer relative tail length in females (TaL/TL 6.1% vs 3.9–4.7%), yellowish-white, immaculate ventrals (vs light orange to salmon with small black spots).

It is distinguished from *Calamaria arcana* (Guangdong, Fujian, and Hunan provinces, possibly occurring in Zhejiang Province, China) by having lower ventral scale counts in both sexes (VEN 145–155 in males and 168 in females vs 170–176 in males and 192 in females), slightly higher subcaudal counts in females (SC 15 vs 12), a smaller total length in males (maximum TL 187 mm vs 304 mm), a slightly longer relative tail length in females (TaL/TL 6.1% vs 4.7%), fewer maxillary teeth (MT 8 vs 10).

*Calamaria similis* sp. nov. further differs from *C. berezowskii* (Sichuan Province, China) by having a smaller total length in both sexes (maximum TL 187 mm in males and 154 mm in females vs 290 mm in males and 245 mm in females); a broad and distinct nuchal collar (vs narrow and indistinct); and presence of two pairs of light spots on the dorsal tail (vs absent).

It can be distinguished from *Calamaria jinggangensis* (Jiangxi, Guizhou, and possibly Hunan provinces, China) by having slightly lower ventral scale counts in both sexes (VEN 145–155 in males and 168 in females vs 157–158 in males and 179 in females), slightly higher subcaudal counts in females (SC 15 vs 12), a shorter relative tail length in males (TaL/TL 6.4–9.3% vs 15%) but a longer relative tail length in females (TaL/TL 6.1% vs 3.6%).

*Calamaria similis* sp. nov. differs from *C. mizoramensis* (Mizoram State, India and Bangladesh) by having a smaller total length in both sexes (maximum TL 187 mm in males and 154 mm in females vs 251 mm in males and 258 mm in females), a shorter relative tail length in males (TaL/TL 6.4–9.3% vs 10.3–13.1%), and presence of two pairs of light spots on the dorsal tail (vs absent).

*Calamaria similis* sp. nov. differs from *C. synergis* (Yunnan Province, China) by having six scales surrounding the paraparietal (vs five), lower ventral scale counts in males (VEN 145–155 vs 161–166), a slightly smaller maximum total length in males (maximum TL 187 mm vs 284 mm), and presence of two pairs of light spots on the dorsal tail (vs absent).

*Calamaria similis* sp. nov. differs from *C. pfefferi* Stejneger, 1901 (Okinawa Island, Japan) by having higher ventral scale counts in females (VEN 168 vs 158–165), fewer subcaudal scales in males (SC 20–23 vs 24–26), presence of a nuchal collar without pattern (vs absent), and presence of two pairs of light spots on the dorsal tail (vs absent) (Stejneger 1907; Maki 1931; Yoshigo et al. 2003; Nakamura et al. 2025).

*Calamaria similis* sp. nov. differs from *C. pavimentata* sensu stricto (Java Island, Indonesia; data based on the combined morphology of the holotypes of *C. pavimentata* and its junior synonym *C. quadrimaculata* following Inger and Marx 1965) by having higher ventral scale counts in females (VEN 168 vs 136), fewer subcaudal scales in males (SC 20–23 vs 27) but slightly higher subcaudal counts in females (SC 15 vs 13), a smaller total length in males (maximum TL 187 mm vs 206 mm), and a shorter relative tail length in both sexes (TaL/TL 6.4–9.3% in males and 6.1% in females vs 11.6% in males and 8.0% in females). Historical illustrations of the holotypes of *C. pavimentata* and its junior synonym *C. quadrimaculata* (Jan and Sordelli 1860–1866) indicate presence of a midventral longitudinal stripe on the tail in *C. pavimentata* and subcaudal blotches in *C. quadrimaculata* (Appendix 1); however, such markings appear to be variable within members of the *C. pavimentata* species complex and may also occur in the new species, and therefore were not used as diagnostic characters here.

Lastly, *Calamaria similis* sp. nov. differs from all five other junior synonyms and subspecies of *C. pavimentata*. Among these, it can be distinguished from *Calamaria siamensis* Günther, 1864 by having a lower maximum ventral scale count (VEN 168 vs 190) and by presence of nuchal collar with no pattern (vs absent) (Günther, 1864). It further differs from Calamaria pavimentata var. uniformis Smith, 1921 by having lower subcaudal scale counts (SC 20–23 in males and 15 in females vs 30–34 in males and 18–19 in females), and by presence of two pairs of light spots on the dorsal tail (vs absent) (Smith 1921). From *Calamaria pavimentata formosana* Maki, 1931, it differs by having slightly lower ventral scale counts in males (VEN 145–155 vs 153–178a smaller maximum total length (max TL 187 mm in males and 154 mm in females vs 280 mm in males and 310 mm in females), and presence of two pairs of light spots on the dorsal tail (vs absent) (Stejneger 1907; Maki 1931). From *Calamaria pavimentata banaensis* Bourret, 1934, it differs by having lower ventral scale counts in both sexes (VEN 145–155 in males and 168 in female vs 157–160 in males and 178–179 in females), lacking a dark midventral stripe on the belly (vs present), and by presence of two pairs of light spots on the dorsal tail (Bourret 1934). Finally, *Calamaria similis* sp. nov. differs from *Calamaria pavimentata miyarai* Takara, 1962 by having lower ventral scale counts in males (VEN 145–155 vs 157–174), fewer subcaudal scales in females (SC 15 vs 17–19), a smaller maximum total length (187 mm in males and 154 mm in females vs 290 mm in males and 368 mm in females), a shorter relative tail length in females (TaL/TL 6.1% vs 7.4–7.6%), and presence of two pairs of light spots on the dorsal tail (Takara 1962; Ota 1982; Ota and Hokama 1996).

##### Distribution, natural history notes, and conservation status.

*Calamaria similis* sp. nov. had long been misidentified as *Calamaria pavimentata*. However, due to the lack of available molecular material, its confirmed distribution is currently restricted to Yangchun City, Yangjiang City, Guangdong Province, and Longzhou County, Chongzuo City, Guangxi ZAR, China. All specimens were collected during the daytime. The holotype (SYS r001816) was found beneath a thick leaf-litter layer in a hilly landscape, whereas two paratypes (SYS r001725 and SYS r001726) were encountered along a roadside in limestone karst forest. Given its semi-fossorial habits, the species is currently known from only a few collected specimens from Guangdong Province and Guangxi ZAR, China, and a small number of photographic records. Notably, the straight-line distance between the confirmed localities in Guangdong Province and Guangxi ZAR exceeds 450 km, encompassing extensive areas that remain unsurveyed. Virtually nothing is known about its population size, ecology, or precise distributional limits. Given this paucity of information, we recommend that *Calamaria similis* sp. nov. be provisionally assessed as Data Deficient (DD) under the IUCN Red List criteria (IUCN Standards and Petitions Subcommittee 2025).

## Discussion

### Taxonomic implications within the *Calamaria pavimentata* complex

The recognition of *Calamaria similis* sp. nov. provides further support for the view that *C. pavimentata* sensu lato represents a complex of morphologically similar but evolutionarily distinct species rather than a single, widely distributed taxon. Historically, the name *C. pavimentata* has been applied broadly to populations spanning southern China, mainland Southeast Asia, northeastern India, eastern Bangladesh, Java (Indonesia), and the Malay Peninsula, largely on the basis of overall similarity in body form and scalation. However, recent integrative taxonomic studies have demonstrated that many of these populations correspond to distinct lineages that warrant species-level recognition, including *C. annamensis*, *C. arcana*, *C. berezowskii*, *C. mizoramensis*, and *C. synergis*.

Within this context, *Calamaria similis* sp. nov. represents the southern Chinese lineage previously referred to as *C. pavimentata* (Yang and Zheng 2018; Yeung et al. 2022). Consistent differences in ventral and subcaudal scale counts, body size, and tail proportions, together with its distinct phylogenetic placement based on mitochondrial DNA data, strongly support its recognition as a separate species. These findings further underscore that reliance on generalized external resemblance has historically obscured substantial cryptic diversity within the *C. pavimentata* species complex.

More broadly, several species of *Calamaria* are characterized by narrow ranges of endemism, such that superficially similar populations may represent distinct evolutionary lineages across multiple species complexes, including those traditionally referred to *C. lumbricoidea* and *C. septentrionalis*. Earlier syntheses, most notably the influential monograph of Inger and Marx (1965), relied primarily on external morphology and adopted broad species concepts that likely underestimated the true diversity within the genus. As a result, the name *Calamaria pavimentata* has been applied widely and inconsistently across Asia, potentially masking multiple distinct species under a single nominal taxon. Accordingly, all nominal taxa and putative synonyms historically placed under *C. pavimentata* should be re-assessed using integrative approaches that combine detailed morphological reexamination of name-bearing types with molecular data from topotypic or near-topotypic populations whenever possible. Likewise, published records of *C. pavimentata* from China warrant critical reevaluation, as they may represent a mixture of distinct taxa rather than *C. pavimentata* sensu stricto.

Historical records from Guangxi ZAR, China, further illustrate this taxonomic complexity. Fan (1931) reported *Calamaria pavimentata* from Jinxiu County, Laibin City, based on two specimens. Although these specimens are no longer extant and cannot be re-examined, the original data indicate notable differences in meristic characters when compared with *Calamaria similis* sp. nov. In particular, the reported male specimen exhibits a markedly higher ventral scale count than that observed in male *C. similis* sp. nov., suggesting that the Jinxiu population may not be conspecific with the new species described herein. In the absence of voucher material and molecular data, however, the taxonomic identity of this population remains unresolved, highlighting the need for renewed field surveys and the collection of new material from this region.

### Population divergence within *Calamaria similis* sp. nov.

Mitochondrial DNA analyses recovered two geographically structured lineages within *Calamaria similis* sp. nov., corresponding to populations from Guangdong Province and Guangxi ZAR, China, with an uncorrected *p*-distance of 10.78% in the cyt *b* gene. Although this level of mitochondrial divergence is relatively high for populations currently assigned to the same species, the two localities are separated by approximately 350 km, and no consistent or diagnostic morphological differences were detected between the two populations. All examined specimens are morphologically indistinguishable with respect to scalation, body proportions, coloration, and osteological characters, despite occurring in markedly different substrates, with the Guangdong population inhabiting soil-based hilly landscapes and the Guangxi population associated with limestone karst environments. Comparable or even higher levels of intraspecific mitochondrial divergence have been reported in other species of *Calamaria* and in fossorial snakes more broadly and are commonly interpreted as the result of long-term population isolation and limited dispersal rather than species-level differentiation. Given the limited sample size and the absence of corroborating morphological differentiation, we therefore regard the Guangdong and Guangxi populations as conspecific and refer them to a single species, *Calamaria similis* sp. nov. Nevertheless, additional sampling from intervening areas, together with analyses incorporating nuclear genetic markers, will be essential to further assess population structure and to test for the possible presence of cryptic diversity within this species.

### On the identity and distribution of *Calamaria pavimentata* sensu stricto

*Calamaria pavimentata* was originally described from Java Island, Indonesia, yet the validity of this type locality has long been questioned, as no unequivocal modern records of the species from Java are currently known. Despite this uncertainty, the name has historically been applied to geographically distant populations without rigorous comparative assessment. It should also be noted that the historical type locality of “Java” may itself be uncertain. During the nineteenth century, specimens were frequently shipped to Europe through major trading ports such as Java, and locality information associated with museum specimens was sometimes generalized or lost during subsequent curation. As a result, it is possible that the type specimens of *C. pavimentata* and its nominal synonym *C. quadrimaculata* did not originate from Java in the strict sense. This possibility should be considered when interpreting the historical distribution and synonymy of taxa associated with the *C. pavimentata* species complex. Nevertheless, the scalation and body proportions reported for *Calamaria pavimentata* sensu stricto in the original description and subsequent accounts differ from those observed in the currently recognized members of the *C. pavimentata* species complex, including the new species described herein. The absence of molecular data from topotypic material or confidently identified Javan populations remains a major impediment to resolving the identity of *C. pavimentata* sensu stricto. However, the substantial morphological differences observed between *Calamaria similis* sp. nov. and the type species of *C. pavimentata*, together with the pronounced geographic disjunction between Java Island and southern China, strongly argue against their conspecificity. When considered alongside the recent recognition of multiple distinct taxa previously subsumed under *C. pavimentata*, it is increasingly likely that the true distribution of *C. pavimentata* sensu stricto is far more restricted than traditionally assumed. Targeted field surveys in Java are therefore urgently needed to rediscover this taxon and to obtain fresh material suitable for molecular analyses. Where feasible, future studies may also explore the potential of historical DNA techniques applied to the type specimen to clarify the taxonomic identity of *C. pavimentata* sensu stricto.

### Biogeographic considerations

The discovery of *Calamaria similis* sp. nov. is consistent with broader biogeographic patterns observed in Southeast Asian and southern Chinese herpetofauna (de Bruyn et al. 2014; Poyarkov et al. 2021, 2023). Considerable phylogenetic structuring between Sundaic island lineages and mainland Asian taxa has been documented across numerous reptile groups and is commonly attributed to long-term geographic isolation and historical landmass fragmentation (e.g., O’Connell et al. 2018). The placement of *Calamaria similis* sp. nov. within a mainland Asian clade of *Calamaria* is concordant with this pattern. Similar mainland–Sundaland phylogeographic breaks have been reported in other fossorial or semi-fossorial reptiles (e.g., Quah et al. 2020). Southern China is increasingly recognized as a center of endemism for small, fossorial reptiles, including several recently described species of *Calamaria* (see Yang and Zheng 2018; Yeung et al. 2022; Zhang et al. 2025a; this study). The restricted and patchy distribution currently known for *Calamaria similis* sp. nov. suggests that additional undescribed diversity may persist in this region, particularly in under-surveyed habitats such as limestone karst forests and lowland evergreen forests.

### Morphological variation and species delimitation in *Calamaria*

Species delimitation within *Calamaria* is notoriously challenging due to substantial intraspecific variation in many traditional diagnostic characters. Although several morphological features, such as head scale configurations, scale fusions, and dorsal scale reductions, can be taxonomically informative in some taxa, many species exhibit overlapping conditions in these traits. Recent studies have demonstrated that subtle but consistent differences in quantitative traits, such as ventral and subcaudal scale counts, relative tail length, and body size, can provide valuable taxonomic information when evaluated within an integrative framework (see Yeung et al. 2022; Liang et al. 2024; Zhang et al. 2025a; Korolev et al. 2026; Lalremsanga et al. 2026). In *Calamaria similis* sp. nov., these differences are stable across the examined specimens and are congruent with mitochondrial DNA divergence, reinforcing their taxonomic significance. Our findings further emphasize that minor morphological differences should not be dismissed a priori in *Calamaria*, particularly when they are associated with clear geographic structure and independent molecular evidence. Continued integrative taxonomic work, incorporating broader geographic sampling and multilocus datasets, will be essential for resolving species boundaries and uncovering hidden diversity within this genus.

## Supplementary Material

XML Treatment for Calamaria similis

## Figures and Tables

**Figure A1.**
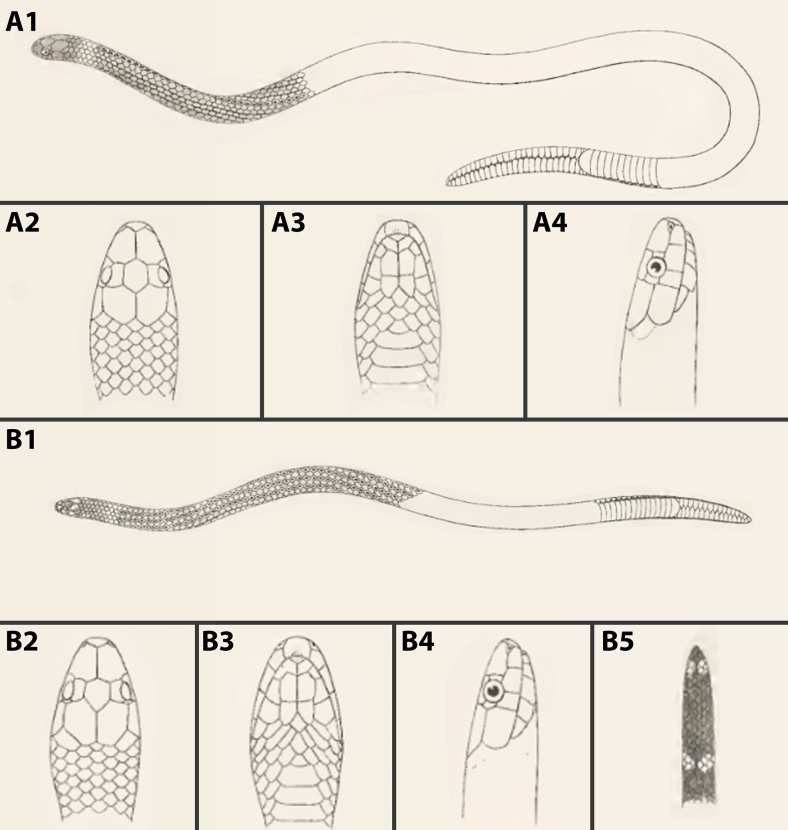
Redrawn figures of the holotypes of *Calamaria pavimentata* Duméril, Bibron & Duméril, 1854 and its junior synonym *C. quadrimaculata* Duméril, Bibron & Duméril, 1854, modified from Jan and Sordelli (1860–1866: Livraison 10, pl. 1). **A**. Holotype of *C. pavimentata* (MNHN-RA-0.3298); **B**. Holotype of *C. quadrimaculata* (MNHN-RA-0.3297); **A1, B1**. Habitus; **A2, B2**. Dorsal view of head; **A3, B3**. Ventral view of head; **A4, B4**. Lateral view of head; **B5**. Ventral view of tail.

## Additional information

### Artificial Intelligence (AI) use

The authors accept full responsibility for the content of the manuscript, including the disclosure of any use of AI.

No AI tools were used in the preparation of this manuscript.

### Author ORCIDs

S. Qi https://orcid.org/0000-0002-2924-6093

T.V. Nguyen https://orcid.org/0000-0001-5413-968X

J-H. Yang https://orcid.org/0000-0002-3706-5380

Y-H. Xu https://orcid.org/0000-0001-6094-6680

P. David https://orcid.org/0000-0001-7562-684X

J-S. Shi https://orcid.org/0000-0001-9168-1734

J-J. Liu https://orcid.org/0009-0006-7002-435X

C-Z. Rong https://orcid.org/0000-0002-7595-6950

A.M. Korolev https://orcid.org/0009-0002-9850-3281

N.A. Poyarkov https://orcid.org/0000-0002-7576-2283

Y-Y. Wang https://orcid.org/0000-0003-2024-6641

### Data availability

All morphological data are provided in Tables 4, 5; DNA sequences are deposited in GenBank.
